# Reprogramming an RNA-guided archaeal TnpB endonuclease for genome editing

**DOI:** 10.1038/s41421-023-00615-2

**Published:** 2023-11-11

**Authors:** Ying Xu, Tao Liu, Jing Wang, Binyang Xiong, Ling Liu, Nan Peng

**Affiliations:** 1 National Key Laboratory of Agricultural Microbiology, Hubei Hongshan Laboratory, College of Life Science and Technology, Huazhong Agricultural University, Wuhan, Hubei China; 2 Shenzhen Institute of Nutrition and Health, Huazhong Agricultural University, Shenzhen, Guangdong China; 3 Shenzhen Branch, Guangdong Laboratory for Lingnan Modern Agriculture, Genome Analysis Laboratory of the Ministry of Agriculture, Agricultural Genomics Institute at Shenzhen, Chinese Academy of Agricultural Sciences, Shenzhen, Guangdong China

**Keywords:** Molecular biology, Biological techniques

Dear Editor,

CRISPR-Cas systems are prokaryotic immune systems, protecting prokaryotes against invasive viruses and plasmids^1^. The CRISPR ribonucleoproteins (RNPs) recognize DNA or RNA target through a mature CRISPR RNA and cleave the target in a sequence-specific manner^2^. The discovery of the CRISPR-Cas system has promoted the development of new genome editing tools based on Cas9^3^ and Cas12a^4^. Recently, new programmable endonucleases have been characterized to expand the genome engineering toolbox. The ancestors of Cas9 and Cas12 family proteins, IscB and TnpB, respectively, have been identified from the widespread IS200/IS605 and IS607 transposon families^5–7^. Both IscB and TnpB use a single noncoding RNA for RNA-guided cleavage of double-stranded (ds) DNA^8,9^, exhibiting genome editing activity in human cells^9,10^.

CRISPR-Cas systems are much more prevalent in archaea, especially in hyperthermophilic archaea, than they are in bacteria^1^. However, almost all the archaea lack class 2 CRISPR-Cas systems^1^, limiting the discovery of new genome editing nucleases from the archaea domain. Considering that transposon families IS200/IS605 encoding TnpB protein are widely present in thermophilic archaea including *Sulfolobales*^11^, we investigated the functions of TnpB from *Sulfolobus islandicus* (SisTnpB1) in this study.

We found that both typical and short IS200/IS605 transposons encoded *tnpA* and *tnpB* genes or encoded *tnpB* gene alone in *S. islandicus* REY15A (Fig. 1a). The phylogenetic tree analysis showed that TnpB associated with TnpA, solo TnpB, and TnpB from *Deinococcus radiodurans* ISDra2^9^ formed 3 distinct branches, suggesting their evolutionary difference (Fig. 1a). According to the previous study^9^, we speculated that the transposon-associated motif (TAM) sequence recognized by TnpB proteins might be identical to the left element (LE) cleavage site-associated motif in *S. islandicus*. We identified conserved DNA sequences at the 5’-end of LE including a conserved AT-rich TAM — 5’-TTTAA-3’, for those carrying both *tpnA* and *tnpB* genes and 5’-TTTAT-3’ for those carrying solo *tnpB* genes by alignment (Fig. 1b; Supplementary Fig. S1a). We also identified that right element (RE) sequences were conserved except their 3’-end sequences (Fig. 1c; Supplementary Fig. S2). The transcripts of these conserved RE sequences containing two inverted repeats close to the 3’-end (Fig. 1c) probably formed two hairpin motifs as the part of ωRNA scaffold (Supplementary Fig. S3a). The non-conserved sequences were predicted to be the guide sequences (Fig. 1c).

**Fig. 1:**
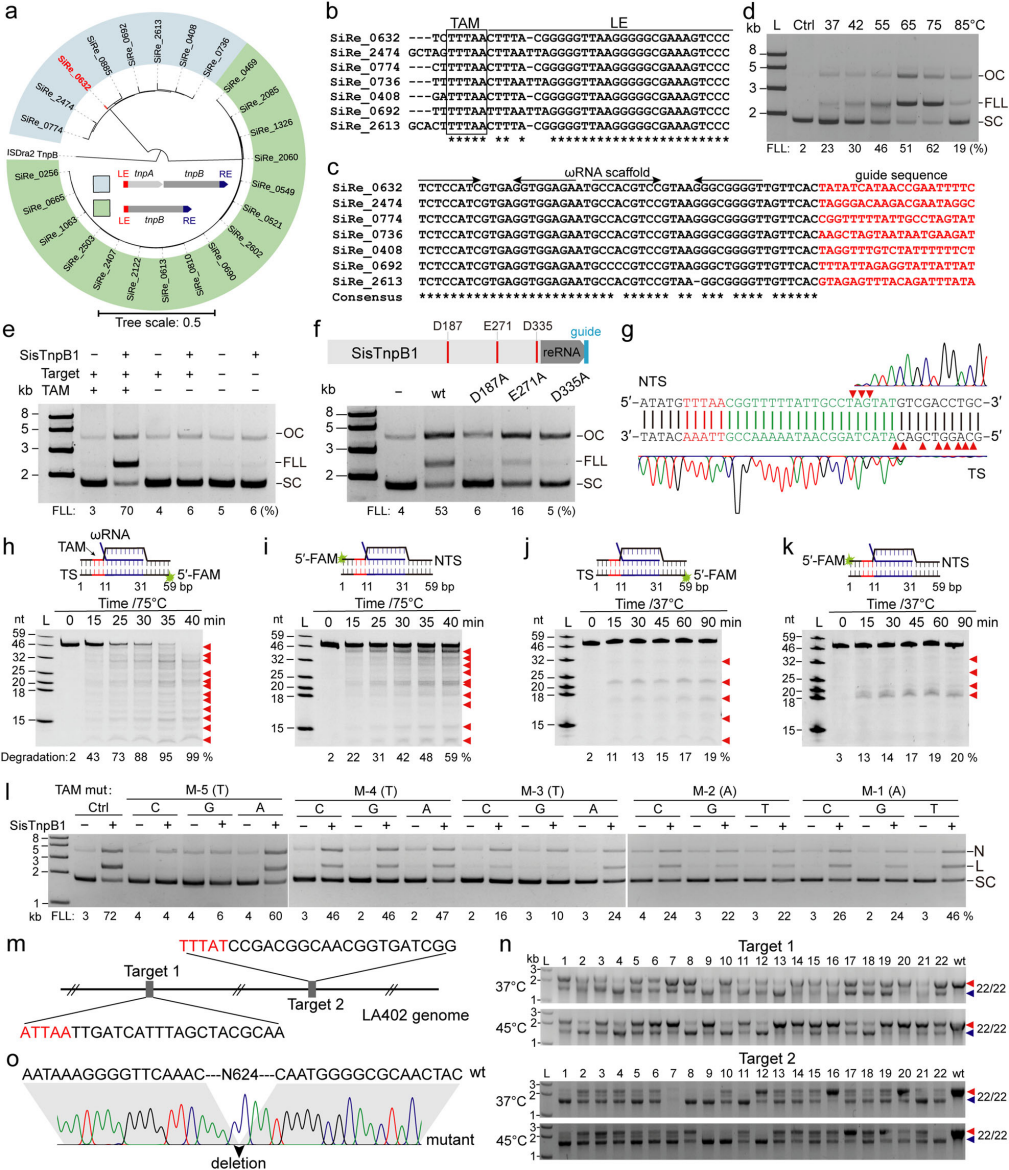
Reprogramming an archaeal TnpB for genome editing. **a** Phylogenetic tree of TnpB proteins from *S. islandicus* REY15A and *D. radiodurans* ISDra2. LE and RE: left or right element flanking the transposon. **b** Predicated TAM sequence (boxed) associated with LE. **c** Alignment of putative ωRNA gene located at the 3’-end of *tnpB* genes. **d** Agarose gel analysis of plasmid DNAs cleaved by SisTnpB1 RNP. Plasmids carrying a 5’-TTTAA-3’ TAM and guide RNA-matching 20-bp target DNA sequence was used as cleavage substrate. OC, open-circle; FLL, full-length linear; SC, supercoiled. **e** Plasmid cleavage by the SisTnpB1 RNP complex in presence or absence of TAM or target sequence at 75 °C. **f** Plasmid cleavage by RNP complexes containing SisTnpB1 mutants with mutations in the predictive RuvC domain (D187, E271 or D335) at 75 °C. **g** Run-off sequencing of plasmid products cleaved by SisTnpB1. Red triangles: cleavage sites on targeted strand (TS) or non-targeted strand (NTS). **h**–**k** 20% denaturing PAGE analysis of 59-bp target dsDNA carrying TAM cleaved by SisTnpB1 RNP at 75 °C (**h**, **i**) or 37 °C (**j**, **k**). TS (**h**, **j**) or NTS (**i**, **k**) were FAM-labeled at 5’ends. Numbers below the diagram indicate the length of the target or matching region between the guide and the target. **l** Agarose gel analysis of the SisTnpB1 RNP-cleaved plasmid DNA with saturation mutations at the 5’-TTTAA-3’ TAM sequence at 75 °C. Numbers are relative to the first nucleotides of the target sequence. Quantitation of FLL cleavage products (**e**, **f** and **l**) and cleavage products (**h**–**k**) were indicated below the lanes. **m** Two selected targets in *pyrE* gene of *P. acidilactici* LA412 genome. Black letters, target sequences; red letters, TAM sequences. **n** Agarose gel analysis of PCR products from 22 randomly selected transformants after one round of passage at 37 °C or 45 °C. Red and blue triangles, wildtype or deletion bands; L, DNA ladder; wt: wildtype cell control. **o** Sanger sequencing of PCR products of the *pyrE* deletion mutant carrying the editing plasmid targeting site 1. The black triangle indicates the site of a 624-bp deletion.

We cloned SiRe_0632 *tnpB* gene and its ωRNA coding sequence into *Escherichia coli* pET30a vector and replaced the putative guide with a sequence matching the target DNA. The purified SiRe_0632 TnpB (named as SisTnpB1) RNP (Supplementary Fig. S3b) was used for in vitro DNA cleavage assay. We found that SisTnpB1 RNP cleaved the supercoiled (SC) target plasmid DNA with a 5’-TTTAA-3’ TAM into full-length linear (FLL) DNA and open-circle (OC) plasmid within a broad range of temperatures, favoring 75 °C (Fig. 1d). Quantitative analysis showed that 23 and 62% FLL DNA products were produced at 37 °C and 75 °C, respectively (Fig. 1d). Additionally, SisTnpB1 RNP cleaved plasmid DNA only in presence of the TAM and target sequence (Fig. 1e). Moreover, SisTnpB1 cleaved plasmid DNA in the presence of Mg^2+^, Mn^2+^ or Ca^2+^ at 37 °C with Mn^2+^ being the most effective metal ion (Supplementary Fig. S3c).

The RuvC-like domain (including residues D187, E271 and D335) was found in SisTnpB1 amino acid sequence (Fig. 1f; Supplementary Fig. S4). Mutation at either D187, E271 or D335 strongly compromised cleavage activity (Fig. 1f). Run-off sequencing of cleavage products showed a staggered cleavage pattern at 15–18 nt from the TAM on the non-targeting sequence and at 20–28 nt from the TAM on the targeting sequence (Fig. 1g), resulting in 5’ overhangs.

Further, SisTnpB1 also cleaved linear target dsDNA at 75 °C (Fig. 1h, i). PAGE analysis showed that the target strand (TS) was completely degraded (Fig. 1h), while the non-target strand (NTS) was only partially cleaved (Fig. 1i). At 37 °C, the cleavage of both TS and NTS sequences was dramatically reduced (Fig. 1j, k). SisTnpB1 showed very weak cleavage activity at TS, and no cleavage at NTS in absence of a TAM sequence at 75 °C (Supplementary Fig. S5a, b), and almost no cleavage activity at 37 °C (Supplementary Fig. S5c, d). SisTnpB1 also cleaved a matched single-stranded (ss) DNA at 75 °C or 37 °C with or without the presence of TAM (Supplementary Fig. S5e–h). The presence of a TAM sequence in ssDNA substrate resulted in higher cleavage efficiency (Supplementary Fig. S5e, g). Lastly, SisTnpB1 showed no cleavage activity on the dsDNA (Supplementary Fig. S5i, j) or ssDNA without a target DNA sequence (Supplementary Fig. S5k, l).

The effects of seed and TAM sequence variation on SisTnpB1 endonuclease activity were studied (Supplementary Fig. S6a). Mutation M1 ~ 5 represented transversion of +1GCCAA + 5 into +1CGGTT + 5 on the target sequence, and this mutation almost abolished DNA cleavage (Supplementary Fig. S6b). Similarly, mutation M6 ~ 10 drastically reduced DNA cleavage, and M11 ~ 15 and M16 ~ 20 had weak effect on target DNA cleavage (Supplementary Fig. S6b), indicating that the seed sequence was located at the site from +1 to +10. Moreover, M–1 ~ –5 with transversion mutation of the TAM sequence almost eliminated DNA cleavage by SisTnpB1 (Supplementary Fig. S6b). Transversion mutation at +1 nucleotide strongly inhibited SisTnpB1 cleavage, while single transversions at other sites had less effect (Supplementary Fig. S6c). Besides, transversion and transition mutations at any nucleotide on the TAM sequence had weak effect on the target DNA cleavage by SisTnpB1 (Fig. 1l), except for the mutant M–5 (T to C), M–5 (T to G) and M–3(T to G) (Fig. 1l). These results indicated that SisTnpB1 was tolerant to mutations on the target DNA sequence and required a less conserved TAM sequence (5’-WNHNN-3’).

The efficiencies of in vivo DNA interference and genome editing were studied in *Pediococcus acidilactici* LA412, which grows well from 37 °C to 55 °C (Supplementary Fig. S7a). We selected a 20-bp target sequence with a 5’-end TTTAA sequence, which was present in both GE00014 gene of plasmid 1 and GE00039 gene of plasmid 2^12^. We cloned ωRNA sequence with a guide matching to the above targets and *SistnpB1* gene into the plasmid pMG36e to obtain an interference plasmid (Supplementary Fig. S7b). The target region on GE00014 gene on plasmid 1 and GE00039 gene on plasmid 2, or GE00037 gene on plasmid 1 and GE00033 gene on plasmid 2 of these transformants carrying the interference plasmid cultured at 37 °C was PCR amplified. The agarose gel analysis showed that the bands of PCR products were weaker than those of the wildtype control (Supplementary Fig. S7c). Importantly, after one-round passage of cells in modified MRS medium containing erythromycin, no bands of PCR products of the target regions were detected (Supplementary Fig. S7c), indicating that the endogenous plasmids were depleted through DNA cleavage by the plasmid-encoded SisTnpB1 RNP.

In order to establish the genome editing tool based on SisTnpB1, we selected two target sites respectively adjacent to 5’ ATTAA and 5’ TTTAT motif on *pyrE* gene (Fig. 1m). The pMG36e-based editing plasmids carrying *SistnpB1* gene, the ωRNA coding sequence with a guide matching to target site 1 or 2 and the repair donor DNA were electroporated into *P. acidilactici* LA412 cells to obtain transformants. The target regions of the transformants were PCR amplified, and the agarose gel analysis indicated that genome editing efficiency at target site 1 of the transformants carrying desired gene deletion was 36.4% and 54.5% at 37 °C and 45 °C, respectively (Supplementary Fig. S7d), and that at target site 2 was 95.5 % and 90.9 %, respectively (Supplementary Fig. S7e). After one-round passage in the antibiotic medium, the genome editing efficiency increased to 100 % for both target sites 1 and 2 at 37 °C or 45 °C (Fig. 1n). Moreover, we found that, from the high-throughput sequencing data, 71.62 % and 82.22 % of reads of the PCR products represented an accurate 624-bp deletion at *pyrE* gene through homology-directed repair in the transformants culturing at 37 °C (Supplementary Fig. S7f). No indels were identified in this experiment, suggesting a deficiency of end-joining pathways in *P. acidilactici*. Sanger sequencing results also verified a 624-bp deletion on *pyrE* gene (Fig. 1o; Supplementary Fig. S7g). After a second- or third-round passage, we could isolate the pure target gene deleted strains (Supplementary Fig. S7h).

Indeed, programmable endonucleases from thermophiles are required as the RNPs since they are very stable and easy to be delivered into the target tissue or bloodstream of the patients or organisms^13^. Although thermo-stable Cas9 nucleases have been identified from bacteria, the optimal reaction temperature for all these CRISPR endonucleases is below 70 °C, except for IgnaviCas9^14^. Our data showed that SisTnpB1 is active between 37 °C and 85 °C (Fig. 1d). Moreover, programmable endonucleases with smaller protein size are required for genome editing. The smaller Cas12f (also known as Cas14) was identified; however, it is a ssDNA-targeting CRISPR endonuclease^15^. SisTnpB1, similar to ISDra2 TnpB and AmaTnpB^8,9^, is 401 amino acid residues in length, which is much smaller than Cas9 and Cas12a endonucleases. Importantly, SisTnpB1 exhibited high editing efficiencies almost up to 100 % in bacterial cells at 37 °C (Fig. 1n) or archaeal cells at 75 °C, suggesting its potential broad applications for different organisms living at different temperatures.

### Supplementary information

Supplementary Informations

## Data Availability

High-throughput sequencing data were deposited in the SRA database under accession number PRJNA999174.
