# Supplementary material for: Reprogramming an RNA-guided archaeal TnpB endonuclease for genome editing
Source: Cell Discov. 2023 Nov 11;9:112. doi: 10.1038/s41421-023-00615-2 (PMC10640517; doi:10.1038/s41421-023-00615-2)
Supplement: Supplementary file 1 — Supplementary Informations [file 41421_2023_615_MOESM1_ESM.pdf]

Reprogramming an RNA-guided archaeal TnpB endonuclease for genome editing

Ying Xu[1,2,3], Tao Liu[1], Jing Wang[1], Binyang Xiong[1], Ling Liu[1] and Nan Peng[1,2,3]*

[1] National Key Laboratory of Agricultural Microbiology, Hubei Hongshan Laboratory, College of Life Science and Technology, Huazhong Agricultural University, Wuhan, 430070, P. R. China

[2] Shenzhen Institute of Nutrition and Health, Huazhong Agricultural University

[3] Shenzhen Branch, Guangdong Laboratory for Lingnan Modern Agriculture, Genome Analysis Laboratory of the Ministry of Agriculture, Agricultural Genomics Institute at Shenzhen, Chinese Academy of Agricultural Sciences, Shenzhen, China

*Correspondence: Nan Peng (nanp@mail.hzau.edu.cn)

Supplementary Materials and Methods

Strains, culture conditions, and transformation

S. islandicus REY15A was cultured in SCV medium at 78°C [1]. *Pediococcus acidilactici* LA412 was cultured in the modified MRS medium at 37°C [2]. *P. acidilactici* competent cells were prepared and electroporated, as described previously [2]. *E. coli* DH5α was used for DNA cloning. All *E. coli* strains were cultured in Luria-Bertani (LB) medium at 37°C, and erythromycin (400 µg/ml) was added when required.

TnpB identification from *S. islandicus*

The genome of *S. islandicus* REY15A was downloaded from NCBI GenBank (Accession No. NC_017276.1). The IS200/IS605 and IS607 transposon families were identified from *S. islandicus* REY15A genome using ISfinder [3]. Then, 24 TnpB family proteins were identified from the IS200/IS605 and IS607 transposon families, and their amino acids sequences were used for phylogenetic analysis. These TnpB protein sequences from *S. islandicus* REY15A were aligned to ISDra2_TnpB (AAF10241.1) using ClustalW with the default settings, and based on alignment results, a phylogenic tree was constructed using MEGA7 (version 7.0.26) software by neighbor-joining method [4], and iTOL was used for downstream visualization of the phylogenic tree [5].

TAM and RE-RNA (ωRNA) analysis

Transposon-associated motif (TAM) sequence has been reported to match the target site sequence of TnpA-mediated transposon [6]. The DNA sequences from -100 to +50, relative to start codon of *tnpA* genes associated with *tnpB* genes and solo *tnpB* genes were extracted for alignment. Next, the first five conserved bases were predicted to be the TAM sequences. To predict the RE-RNA (ωRNA), nucleotide sequences within 250 bp ahead to100 bp behind the stop codon of each *tnpB* gene were extracted for alignment. Based on the selected conserved regions, ωRNA scaffold was predicted using the "UNAFold Web Server" with its default settings

(http://www.unafold.org/DNA_form.php) [7]. The 20 nt sequences located behind the conserved region at the 3'end of the ωRNA were predicted to be the guide sequences.

Plasmid construction

The *SistnpB1* gene (SiRe_0632) and the predicated ωRNA-coding DNA were amplified from *S. islandicus* REY15A genomic DNA. The *SistnpB1* gene PCR product was digested with Nde I/Sal I, purified, and cloned into pET30a to obtain plasmid pET30a-*SistnpB1*. Then, the ωRNA-coding DNA was digested with Not I/Xho I and cloned into plasmid pET30a-*SistnpB1*. DNA oligo pairs (synthesized by Sangon Biotech, Shanghai) were annealed to generate target DNA, and obtained target DNA was cloned into pUC19 plasmid. The inverse PCR was conducted to obtain target plasmids and mutation *SistnpB1* gene plasmids using the primers carrying the mutation sequences. The PCR products were purified, treated with Dpn I, treated with recombinase, and transformed into *E. coli* DH5α cells. The fluorescence oligos (synthesized by Sangon Biotech, Shanghai) were annealed in a thermo-cycler to generate linear DNA targets for subsequent *in vitro* cleavage. *SistnpB1* gene and ωRNA-coding DNAs respectively under control of the *lacZ* gene promoter and P32 promoter were cloned into pMG36e plasmid through T5 exonuclease DNA assembly (TEDA) to obtain plasmid pSisTnpB1-ωRNA. Then, the DNA donors upstream and downstream target sites were PCR amplified from pMG36e-C-LR [2] and cloned into plasmid pSisTnpB1-ωRNA to generate the genome editing plasmid for deletion of *pyrE* gene.

The *SistnpB1* gene, the ωRNA coding sequence targeting *cas6* gene and the donor DNA were cloned into *Sulfolobus* expression vector pSeSD [8], resulting the *Sulfolobus* editing plasmid targeting *cas6* gene. All primers used in this study were presented in Supplementary Table 1.

Expression and purification of SisTnpB1 RNP complex

E. coli BL21-AI cells carrying *SistnpB1*-ωRNA expression cassette were culture in LB medium supplemented with ampicillin (100 μg/ml) and chloramphenicol (50

μg/ml) at 37°C. After culture to an OD_{600} of 0.6–0.8, protein expression was induced with 0.2% arabinose, and the cells were cultured overnight at 16°C. Then, the cells were harvested by centrifugation, resuspended in 20 mM Tris-HCl (pH 8.0) buffer (containing 250 mM NaCl, 5 mM 2-mercaptoethanol, 25 mM imidazole, 2 mM PMSF, and 5% (v/v) glycerol), and disrupted through high pressure. After removing cell debris by centrifugation, the supernatant was loaded onto the Ni^{2+}-charged HiTrap chelating HP column (Cytiva, Marlborough, MA, USA). Subsequently, proteins were eluted with a gradient imidazole (concentration increasing from 25 mM to 500 mM) in the buffer containing 20 mM Tris-HCl (pH 8.0), 500 mM NaCl, 5 mM 2-mercaptoethanol, and 5% (v/v) glycerol. The protein fractions containing SisTnpB1 RNP or the mutant proteins were collected, concentrated through ultrafiltration, and separated through a Superdex 200 Increase 10/300 GL column (Cytiva, Marlborough, MA, USA) using 20 mM HEPES buffer, pH7.0, containing 500 mM NaCl, and the peaks of target proteins were analysed by SDS-PAGE. The samples containing SisTnpB1 RNP were dialysed in 20 mM Tris-HCl buffer (pH 8.0 at 25°C) and stored at -80°C or immediately used.

DNA cleavage assay

Plasmid DNA and synthetic oligoduplex cleavages experiments were conducted at different temperatures (37-85°C) for 60 minutes by adding 5 nM SisTnpB1 RNP complex and 5 ng/μL plasmid DNA carrying different target sequences and TAM sequences in 10 mM Tris-HCl buffer (pH7.5) containing 1 mM DTT, 1 mM EDTA,100 mM NaCl, with 10 mM $MgCl_2$, $MnCl_2$, $CaCl_2$, $ZnCl_2$ or $NiCl_2$ added into buffer for different reactions. The reaction was stopped by adding 20 mM protease K and 4% SDS solution, and then reaction system was incubated at 37°C for 1 hour. Then, 2 × loading dye was added, and cleavage products were subjected to 1.5% agarose gel analysis or 20% denaturing PAGE electrophoresis. DNA fragments in agarose gel were visualized by ethidium bromide staining, and DNA fragments in denaturing PAGE were detected using a FUJIFILM scanner (FLA-5100). Band staining intensity was determined by densitometry, using the software ImageJ.

Genome editing in *P. acidilactici* and *S. islandicus* cells

P. acidilactici colonies carrying the editing plasmids on the plates containing 5 μg/mL erythromycin were selected randomly, transferred to liquid medium containing 5 μg/mL erythromycin, and incubated at 37°C or 45°C for 12 h. These cell cultures were transferred into fresh medium containing 5 μg/mL erythromycin for the second-round incubation. The editing region was PCR amplified, and PCR products were separated on 1.5% agarose gel. The samples containing shorter bands (shown on gel, representing the successful region deletion) were subjected to DNA sequencing. Moreover, *P. acidilactici* colonies on the selection medium plates were washed into fresh medium and cultured for 1 day at 37°C. The washed cell culture or the culture after one round incubation was used as the PCR template to amplify the targeting region. Then, the PCR products were sequenced using the Illumina NovaSeq platform at Shanghai Personalbio Technology and the sequencing data were deposited in the SRA database under Accession PRJNA999174.

Sulfolobus editing plasmid was electroporated into *S. islandicus* E233S cells and colonies carrying the editing plasmid were selected on SCV medium plates [8]. Single colonies were used as PCR templates to amplify the target sites. PCR products were analyzed on 1% agarose gel and the shorter bands were subjected to DNA sequencing.

References

1 Deng, L., Zhu, H., Chen, Z., Liang, Y. X. & She, Q. *Extremophiles* **13**, 735-746 (2009).
2 Liu, L. *et al. Appl Environ Microbiol* **87**, e0094821 (2021).
3 Siguier, P., Perochon, J., Lestrade, L., Mahillon, J. & Chandler, M. *Nucleic Acids Res* **34**, D32-36 (2006).
4 Kumar, S., Stecher, G. & Tamura, K. *Mol Biol Evol* **33**, 1870-1874 (2016).
5 Letunic, I. & Bork, P. *Nucleic Acids Res* **49**, W293-W296 (2021).
6 Karvelis, T. *et al. Nature* **599**, 692-696 (2021).
7 Zuker, M. *Nucleic Acids Res* **31**, 3406-3415 (2003).
8 Peng, N. *et al. Appl Environ Microbiol* **78**, 5630-5637 (2012).

```
a
SiRe_0408         --TATTGTTCTGGTTTTATTCCAGCCCTCACATGGCTTCTTGCCACTA------------
SiRe_2613         ---------------TTATACAACAACTCCTATAATTACGTAATAATACCAAAATGGAGC
SiRe_0774         ----AATTGTTATCTACACTCAG---TTACGATAACTATAATGTTACAC-----------
SiRe_0632         ---------GTGACTTTTTTCTT---CCTCTATACCCATCCTTTCAGCCCATC-------
SiRe_0736         ----------GGATATTATGTGA---ATTAGATAAAGTTTCTCTGAATCAAGAAGATA--
SiRe_2474         -----------------TTTCTA---ACTTTATAAAGAAGTGACTAACAAAGAGAAAAGC
SiRe_0692         AATAGATTTTAGGCGTTGCGTTA---CTTCTCTGCCCTTAATA-----------------
                     TAM                        *    LE
SiRe_0408         -GATTTAACTTTA-CGGGGGTTAAGGGGGCGAAAGTCCCCTTCCGTGA-----GGGATGG
SiRe_2613         ACTTTTAACTTTA-CGGGGGTTAAGGGGGCGAAAGTCCCATTCCGTGA-----GGGATGG
SiRe_0774         -CTTTTAACTTTA-CGGGGGTTAAGGGGGCGAAAGTCCCCTTCCGTGAGGGAGGGGATGG
SiRe_0632         -TCTTTAACTTTA-CGGGGGTTAAGGGGGCGAAAGTCCCCTTCCGTGAGGGAGGGGATGA
SiRe_0736         -TTTTTAACTTAATTAGGGGTTAAGGGGGCGAAAGTCCCCTTCCGTGA-----GGGATGA
SiRe_2474         TAGTTTAACTTAATTAGGGGTTAAGGGGGCGAAAGTCCCCTTCCGTGAGGGAGGGGATGA
SiRe_0692         -TTTTTAATTTAATTAGGGGTTAAGGGGGCGAAAGTCCCCTTCCGTGAGGGAGGGGATGA
                     ***** ** *   *********************** ********     ******

SiRe_0408         ATAGCCCCCTTATAGAAATATTTTTATAGTTATTAAAAAATAGATATAACT +50
SiRe_2613         ATAGCCCCCTTATAGAAACCTTTTTATAGTTATTAAAAAATAGATATAACT +50
SiRe_0774         ATAG-CCCCTTATAGAAACCTTTTTATAGTTATTAAAAAATAGATATAACT +50
SiRe_0632         ATAGCCCCCTTATAGAAACCTTTTTATAGTTATTAAAAAATACATATAACT +50
SiRe_0736         ATAGCCCCCTTATAGAAACCTTTTTATAGTTATTAAAAAATAGATATAACT +50
SiRe_2474         ATAG-CCCCTTATAGAAACCTTTTTATAGTTATTAAAAAATAGATATAACT +50
SiRe_0692         ATAGCCCCCTTATAGAAACCTTTTTATAGTTATTAAAAAATAGATATAACT +50
                  **** *************  ********************** ********

b
SiRe_1326         -------------AAAATAATAATAGGAATATTGCATCCACAATAATGTTAATTATAAGT
SiRe_0665         --------TCTCCCATATATGAGTTCAAACTCATTTTCC--------TCCTCAAAAATCT
SiRe_0810         --------------AAGTACTGCTTCAGTTTCCTCAACGCTTAACCCTTCTAAAGGTTGG
SiRe_1063         -------------TTTAATCCAATCAAAATTT---TTACTGATATTGCAATCTACAAAAG
SiRe_0549         ------------ATCATTAGTAAGTAGTATATGCTCTGTCAGA----CCTCAAAGGGCAA
SiRe_0613         TTGACCAACTTAATTTGTTGAAATGAAAGTAC----------------TTATGGATATTAC
SiRe_2602         ---CCGAGCCATGCTAACTGAGTTCAAAGTAT-------------CGTCATTAGGATAAA
SiRe_0469         --------------GAGAAGTGGAAGAAATGCAGAAGTTGGGAAAATTAATAGAGAGAAT
SiRe_2122         ---------GTAACTATCGGACATTAAGATATATCTTTTTGGAGGCTTTATAAGT-----
SiRe_0690         TAAAAGGGTAGTACCATGTTATATTTAAATA----------------TTGTTATAGTAAT
                    TAM                               LE
SiRe_1326         CCTTTAACTTTACGGGGGTT---AAGGGGGCGAAAGACCCCTTCCGTGAGGGAGGAGATG
SiRe_0665         CATTTATCTTAACAAGGGGTTCAAAGGGGGCGAAAGACCCCTTCCGTGAGGGAGGAGATG
SiRe_0810         AATTTATCTTAACAAGGGGTTCAAAGGGGGCGAAAGACCCCTTCCGTGAGGGAGGAGATG
SiRe_1063         CATTTATCTTAACAAGGGGTTCAAAGGGGGTGAAAGACCCCTTCCGTGAGGGAGTGGATG
SiRe_0549         GATTTATCTTAACAAGGGGTTCAAAGGGGGCGAAAGACCCCTTCCGTGAGGGAGTGGATG
SiRe_0613         TTTTTATCTTAACAAGGGGTTCAAA-GGGGCGAAAGACCCCTTCCGTGAGGGAGTGGATG
SiRe_2602         TGTTTATCTTAACAAGGGGTTCAAAGGGGGCGAAAGACCCCTTCCGTGAGGGAGGGGATG
SiRe_0469         --TTTATCTTAACAAGGGGTTCAAAGGGGGCGAAAGACCCCTTCCGTGAGGGAGGGGATG
SiRe_2122         --TTTATCTTAACAAGGGGTTCAAAGGGGGCGAAAGACCCCTTCCGTGAGGGAGGGGATG
SiRe_0690         GCTTTATCTTAACAAGGGGTTCAAAGGGGGCGAAAGACCCCTTCCGTGAGGGAGGGGATG
                    **** *** **  *** *   ** **** ***********************  ****

SiRe_1326         GATAGCCCCCTTATATTTAAATAATTCTTTTTCAAATATTCTATTA +50
SiRe_0665         GATAGCCCCCTTATATTTAAATAATTCTTTTTCAAATATTCTATTA +50
SiRe_0810         GATAGCCCCCT--TATTTAAATAATTCTTTTTCAAATATTCTATTA +50
SiRe_1063         GATAGCCCCCTTATATTTAAATAATTCTTTTTCAAATATTCTATTA +50
SiRe_0549         GATAGCCCCCTTATATTTAAATAATTCTTTTTCAAATATTCTATTA +50
SiRe_0613         GATAGCCCCCTTATATTTAAATAATTCTTTTTCAAATATTCTATTA +50
SiRe_2602         GATAGCCCCCTTATATTTAAATAATTCTTTTTCAAATATTCTATTA +50
SiRe_0469         GATAGCCCCCTTATATTTAAATAATTCTTTTTCAAATATTCTATTA +50
SiRe_2122         GATAGCCCCCTTATATTTAAATAATTCTTTTTCAAATATTCTATTA +50
SiRe_0690         GATAGCCCCCTTATATTTAAATAATTCTTTTTCAAATATTCTATTA +50
                  ***********  *********************************
```

Supplementary Figure 1. Identification of TAM sequences. The DNA sequences from -100 to +50 relative to the start codon of eight *tnpA* genes associated with *tnpB* **(a)** and solo *tnpB* genes **(b)** were extracted and analyzed through alignment. The first five conserved bases were predicted to be the TAM sequences. TAM sequences are boxed and LE sequences are indicated.

```
SiRe_0692  TTAAGAAGGATTTGACTTTGGCTGACCGTATATTCTCATGTCCTAAATGTGGTTGGACT
SiRe_0774  TTAAGAAGGACTTGACTTTGGCTGACCGTGTGTTTGTATGTCCTAAATGTGGTTGGACT
SiRe_0632  TTAAGAAGGACTTGGCTTTGGCTGACCGTGTGTTTGTATGCCCTGAGTGCGGTTGGACT
SiRe_2474  TTAAGAAGGACTTGGCTTTGGCTGACCGTGTGTTTGTATGCCCCAAGTGTGGTTGGACT
SiRe_0736  TTAAGAAGGACCTGGCTTTGGCTGACCGTGTGTTTGTATGCCCCAAGTGTGGTTGGGCT
SiRe_2613  TTAAGAAGGATTTGACTTTGGCTGACCGTGTGTTTGTATGTCCCAAGTGTGGTTGGACT
SiRe_0408  TTAAGAAGGACTTGGCTTTGGCTGACCGTGTGTTTGTATGTCCCAAGTGTGGTTGGGCT
           **********  ** ************** * **   *** **  * ** ****** **

SiRe_0692 GTAGATCGTGACTATAATGCTTCTCTAAATATTCTTCATGCGGGGTCGGGACAGCCCTTA
SiRe_0774 GTAGATCGTGACTATAATGCTTCTCTAAATATTCTTCGTGCGGGGTCGGGACTGCCCTTA
SiRe_0632 GTAGATCGTGACTATAATGCTTCTCTAAATATTCTTCGTGCGGGGTCGGGACTGCCCTTA
SiRe_2474 GTAGATCGTGACTATAATGCTTCTCTAAATATTCTTCGTGCGGGGTCGGGACTGCCCTTA
SiRe_0736 GTAGATCGTGACTATAATGCTTCTCTAAATATTCTTCGTGCGGGGTCGGGACAGCCCTTA
SiRe_2613 GTAGACCGTGACTATAATGCTTCTCTAAATATTCTTCATGCGGGGTCGGGACAGCCCTTA
SiRe_0408 GTAGATCGTGACTATAATGCTTCTCTAAATATTCTTCATGCGGGGTCGGGACAGCCCTTA
          ***** ******************************* ************** *******

SiRe_0692 GAGCCTGTGGACAGGAGACCTCTGCTATACATTCCCTTCTCTGAGGGTGTGTATAGTAAG
SiRe_0774 GAGCCTGTGGACAGGGGACCTCTGCTATACATTCCCTTCTCAGAAGGGGTGTATAGTAAG
SiRe_0632 GAGCCTGTGGACAGGGGACCTCTGCTATACATTCCCTTCTCTGAGGGTGTGTATAGTAAG
SiRe_2474 GAGCCTGTGGACAGGGGACCTCTGCTATACATACCCTTCTCTGAGGGTGTGTATAGTAAG
SiRe_0736 GAGCCTGTGGACAGGGGACCTCTGCTATACATACCCTTCTCTGAGGGTGTGTATAGTAGG
SiRe_2613 GAGCCTGTGGACAGGGGACCTCTGCTATACATACCCTTCTCTGAGGGTGTGTATAGTAAG
SiRe_0408 GAGCCTGTGGACAGGGGACCTCTGCTATACATACCCTTCTCTGAGGGTGTGTATAGTAGG
          *************** **************** ******** ** ** ********** *

SiRe_0692 TTTCCTGGAAGAAGCAGGAAATCTCCATCGTGAGGTGGAGATGCCCCGTCCGTAAGGGCT
SiRe_0774 TTTCTTGGAAGAAGCAGGAAATCTCCATCGTGAGGTGGAGATGCCACGTCCGTAAGGGCG
SiRe_0632 TTTCTTGGAAGAAGCAGGAAATCTCCATCGTGAGGTGGAGATGCCACGTCCGTAAGGGCG
SiRe_2474 TTTCTTGGAAGAAGCAGGAAATCTCCATCGTGAGGTGGAGATGCCACGTCCGTAAGGGCG
SiRe_0736 TTTCCTGGAAGAAGCAGGAAATCTCCATCGTGAGGTGGAGATGCCACGTCCGTAAGGGCG
SiRe_2613 TTTCCTGGAAGAAGCAGGAAATCTCCATCGTGAGGTGGAGATGCCACGTCCGTAA-GGCG
SiRe_0408 TTTCCTGGAAGAAGCAGGAAATCTCCATCGTGAGGTGGAGATGCCACGTCCGTAAGGGCG
          **** **************************************** ********* ***

SiRe_0692 GGGTTGTTCACTTTATTAGAGGTATTATTAT
SiRe_0774 GGGTTGTTCACCGGTTTTTATTGCCTAGTAT
SiRe_0632 GGGTTGTTCACTATATCATAACCGAATTTTC
SiRe_2474 GGGTAGTTCACTAGGGACAAGACGAATAGGC
SiRe_0736 GGGTAGTTCACAAGCTAGTAATAATGAAGAT
SiRe_2613 GGGTTGTTCACGTAGAGTTTACAGATTTATA
SiRe_0408 GGGTTGTTCACTAGGTTTGTCTATTTTTTCT
          **** ******    guide sequence
```

Supplementary Figure 2. Identification of ωRNA sequences. The ωRNA sequences located at the 3'-end of *tnpB* genes associated with *tnpA* gene were aligned, and the guide sequences were boxed.

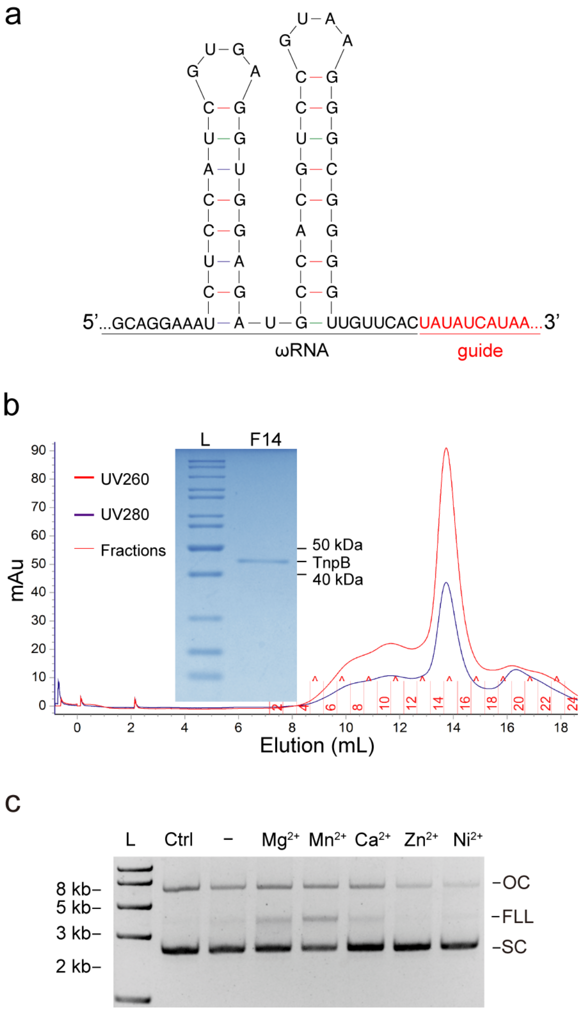

Supplementary Figure 3. *In vitro* DNA cleavage by SisTnpB1 RNP. (a) Predicted structure of ωRNA from the RE of SiRe_0632 *tnpB* gene. (**b**) Purification of SisTnpB1 RNP from *E. coli*. Gel filtration profile of SisTnpB1 RNP and SDS-PAGE analysis of fraction 14 from gel filtration. L: protein ladder; F14, fraction 14. (**c**) metal ion-dependent endonuclease activity of SisTnpB1. DNA cleavage experiment was carried out at 37°C because the presence of Mn^{2+} at 75°C caused DNA degradation.

```
ISDra2TnpB      MIRNK--------AFVVRLYPNAAQTELINRTLGSARFVYNHFLARRIAAYKESGKGLTY 52
SisTnpB1        MWRAKEKNDKTQLSFKYKIYPTREVEEKLIKVMQIEAKVYNALLD-VINNARKEGKKITP 59
                * * *        :*  .:**.    * : ..:     *** :*   *   .:.** :*

ISDra2TnpB      GQTSSELTLLKQAEETSWLSEVDKFALQNSLKNLETAYKNFFRTVKQSGKKVGFPRFRKK 112
SisTnpB1        KDTQNMLKELKIEGKELVYSKALQMVNNQLWSNI-----NALHELKKKGKKVGKLRYKKI 114
                 :*.. *. **   :    *:. ::. ::  .*:     * :. :*:.*****  *:.*

ISDra2TnpB      RTGESYRTQFTNNNIQIGEGRLKLPKLGWVKTKGQQDIQGKILNVTVRRIHEGHYEASVL 172
SisTnpB1        LK----IINYNQSGFKVEGDKLILSKIGEIKVLFHRPLEGEIKGVIIKKSSTGWY-AIFQ 169
                 .      ::.:..:::  ..* *.*:* :*.  :. ::*:* .* :..   *.* * .

ISDra2TnpB      CEVEIPYLPAAPKFAAGVDVGIKDFAIVTDGVRFKHEQNPKYYRSTLKRLRKAQQTLSRR 232
SisTnpB1        VEVEKKPLEKAGK-VVGIDLGVDKLVTTSDGVVI---ENPKVFDKVERRIKILQKSLSRK 225
                 ***   *  * * ..*:*:*:..:. .:*** :   :*** : .. .*:.  *::***.

ISDra2TnpB      KKGSARYGKAKTKLARIHKRIVNKRQDFLHKLTTSLVREYEIIGTEHLKPDNM---RKNR 289
SisTnpB1        KKGSRNYEKVRKKLAKLHEHVKNLMSDYIHKVTSWLVEEYDEIYVEDLDVKDIVEDSESK 285
                **** .* *...***.:*:.: *  .*::**:*: ** **: * .* *. .::    :..

ISDra2TnpB      RLALSISDAGWGEFIRQLEYKAAWYGRLVSKVSPYFPSSQLCHDCGFKNPEVKNLAVRTW 349
SisTnpB1        VLRKHILHSDFSKFVSYLSYKAERAGRRVVKVDPR-NTSKTCARCGYVKKDL-ALADRVF 343
                 *   *  :.:.:*:  *.*** . ** * **.*   :*: *  **: : ::  ** *.:

ISDra2TnpB      TCPNCGETHDRDENAALNIRREALVAAGISDTLNAHGGYVR-PASAGNGLRSENHATLVV 408
SisTnpB1        VCPECGWTVDRDYNASLNILR---AGSGLPLEPVDRGPLLYIPFSEGVYSKFLGRSRKSP 400
                .**:** * *** **:*** *   ..:*:.     .*  :  * * *   .  ..:

ISDra2TnpB      -
SisTnpB1        S
```

Supplementary Figure 4. Analysis of ISDra2 TnpB and SisTnpB1 proteins using CLUSTAL multiple sequence alignment. Conserved amino acid residues in the putative RuvC domains were boxed.

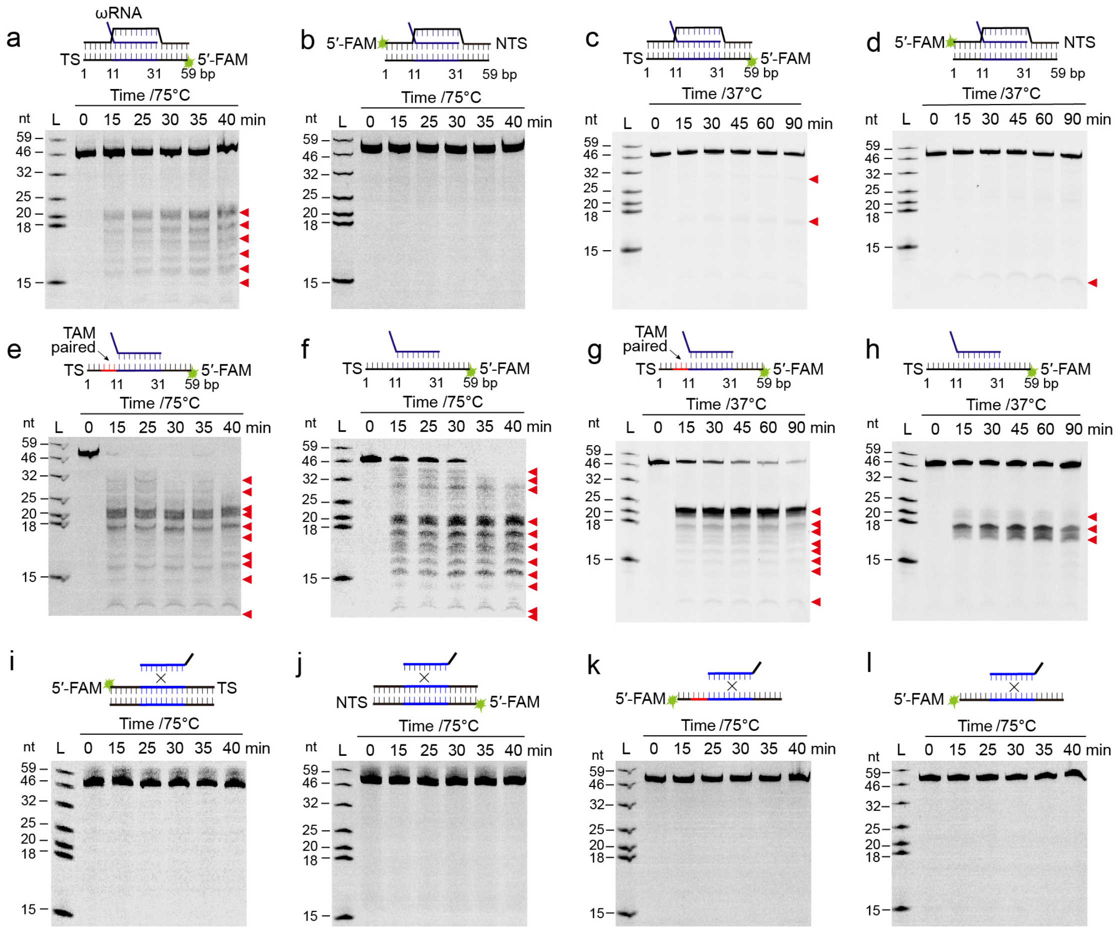

Supplementary Figure 5. SisTnpB1 cleaves both dsDNA and ssDNA. (a - d) 20% denaturing PAGE analysis of 59-bp target dsDNA carrying no TAM cleaved by SisTnpB1 RNP at 75°C **(a** and **b)** or 37°C **(c** and **d)**. **(e - h)** 20% denaturing PAGE analysis of SisTnpB1 RNP-cleaved 59 nt target ssDNA carrying TAM-paired sequence **(e** and **g)** or without TAM **(f** and **h)** at 75°C **(e** and **f)** or 37°C **(g** and **h)**. ssDNAs were FAM-labelled at all 5'ends. Cleaved products were indicated by red triangles. 20% denaturing PAGE analysis of SisTnpB1 RNP-cleaved 59-bp dsDNA (**i** and **j**) or 59-nt ssDNA (**k** and **l**) without matching sequence at 75°C. Target strands **(i, k** and **l)** or non-target strand **(j)** were FAM-labelled at 5'-end. TAM sequence is indicated in red. The matching sites (11 and 39 bp) between the guide of ωRNA and the target as well as the length of the target DNA (59 bp) were labeled.

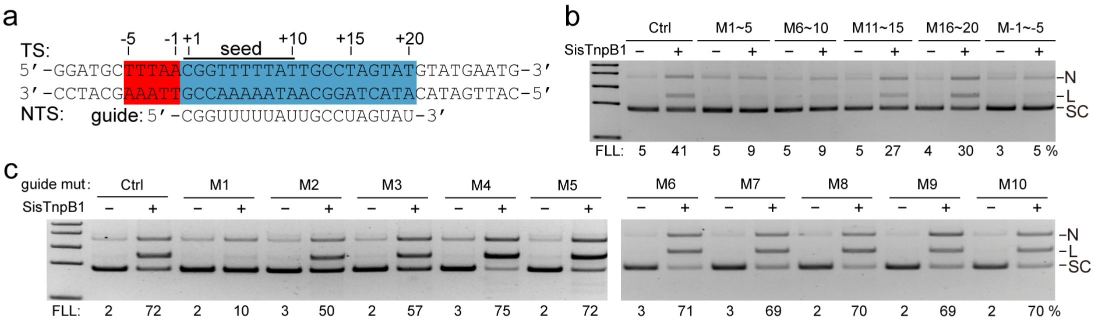

Supplementary Figure 6. Effects of seed and TAM sequence variation on SisTnpB1 endonuclease activity. (a) Schematic of the target dsDNA sequence in the plasmid and guide RNA sequence of the ωRNA. TAM and guide-matching sequences were boxed by red and light blue rectangles, respectively. The location of bases in the TAM and target sequences were labeled by numbers. **(b)** Agarose gel analysis of SisTnpB1 RNP-cleaved products from the substrate plasmid with transversion mutations at the target DNA sequence or TAM sequence. Numbers indicated the mutation regions. **(c)** Agarose gel analysis of the SisTnpB1 RNP-cleaved products from the substrate plasmid with single transversion mutations at the target DNA sequence. Quantitation of FLL cleavage products were indicated below the lanes.

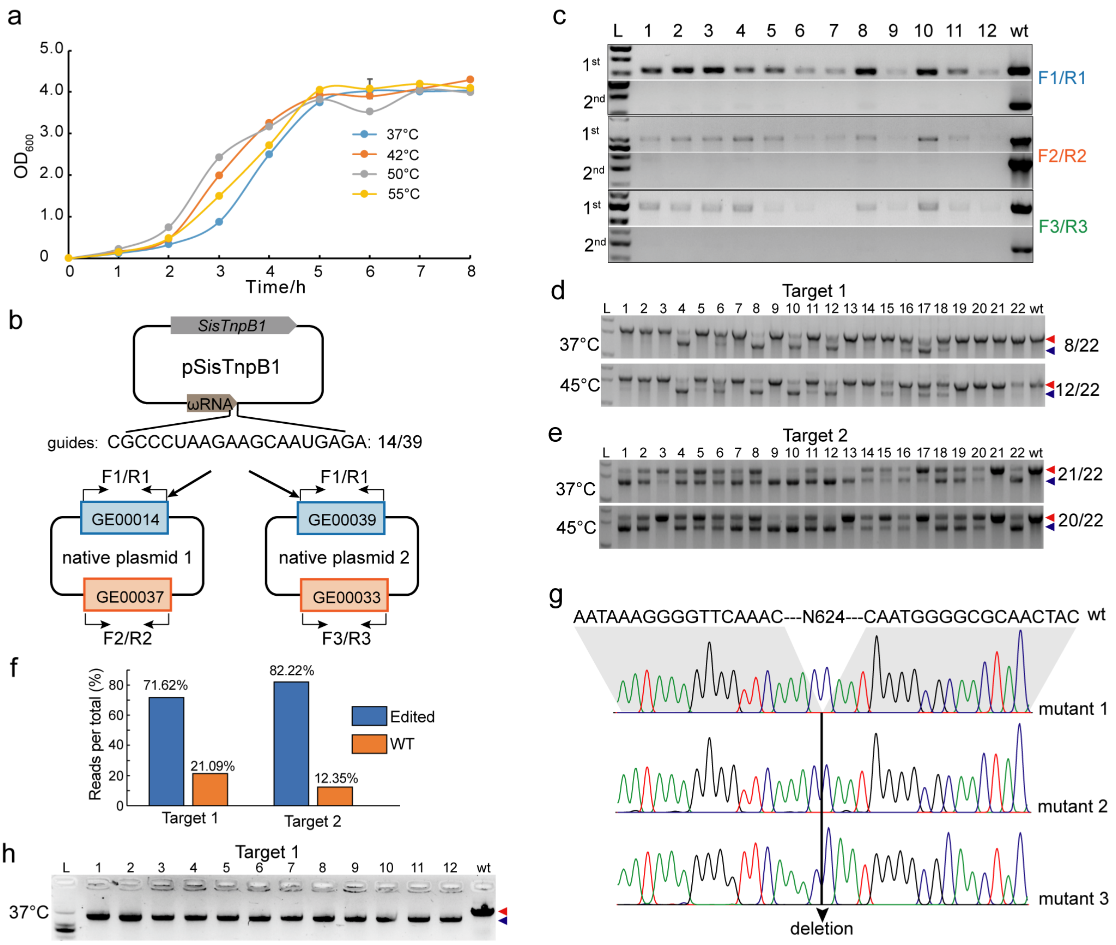

Supplementary Figure 7. Harnessing SisTnpB1 for *in vivo* DNA interference and genome editing in *Pediococcus acidilactici*. (a) Growth curves of *P. acidilactici* LA412 strain at different temperatures in liquid modified MRS medium. Data are expressed as the mean ± SD of three technical replicates. **(b)** Schematic of pMG36e-based plasmid interference. Interference plasmid encodes *SistnpB1* gene and the ωRNA, thus targeting GE00014 of endogenous plasmid 1 and GE00039 gene of endogenous plasmid 2. Primer pairs F1/R1, F2/R2, and F3/R3 were used to amplify GE00014/GE00039 gene, GE00037 gene, and GE00033 gene, respectively. **(c)** Agarose gel analysis of PCR products from randomly selected colonies (1st) or those after one-round passage (2nd). The primer pairs F1/R1, F2/R2, and F3/R3 were used for PCR amplification. Agarose gel analysis of PCR products of the target 1. (**d**) and target 2 (**e**) on *pyrE* gene from randomly selected transformants cultured at 37°C or 45°C. The number on the right of gel bands indicate the editing efficiencies. (**f**) Percentage of the edited reads (an accurate 624-bp deletion) and wildtype (WT) reads per total reads from the PCR products amplifying the target sites of the transformants carrying the editing plasmids after one round passage in antibiotic medium at 37°C. (**g**) Sanger sequencing of PCR products from three more *pyrE* deletion single mutants (mutant 1 to 3) carrying the editing plasmid targeting site 1. The black triangle indicates the site of a 624-bp deletion. (**h**) Agarose gel analysis of PCR products of the target 1 on *pyrE* gene from the colonies spread on MRS plates after a second- or third-round passage of a transformant in the fresh MRS medium containing 5 μg/mL

erythromycin. Red and blue triangles denote wildtype band and deletion band, respectively. L, DNA ladder; numbers on the top of the gels: randomly selected transformants; wt: wildtype cell control.

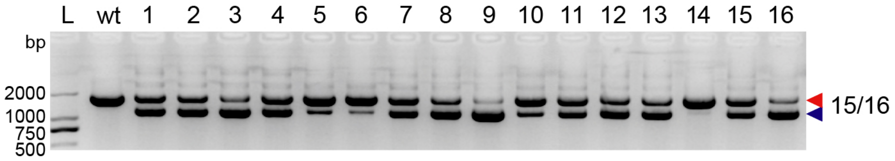

Supplementary Figure 8. Harnessing SisTnpB1 for *cas6* gene deletion in *Sulfolobus islandicus*. Agarose gel analysis of PCR products from randomly selected 16 colonies carrying the editing plasmid. Red and blue triangles denote wildtype band (1764 bp) and deletion band (1162 bp), respectively. L, DNA ladder; numbers on the top of the gels: randomly selected transformants; wt: wildtype cell control.

Supplementary Table 1: primers and oligonucleotides used in this study

Primers and nucleotides	Sequence (5′ to 3′)
**Primers for SisTnpB1 cloning and expression**	
SiRe0632-F-Nde I	CGCCATATGCATCACCATCACCACCATCATCACTGGAGAGCCAA GGAGAAGAATG
SiRe0632-R-Sal I	CGCGTCGACGCTATAACACCAGCTTGCTCAGTTA
ωRNA-F-Not I	ATTGCGGCCGCTAATACGACTCACTATAGGGTTAAGAAGGACTT GACTTTG
ωRNA-R	GTCGACATACTAGGCAATAAAAACCG
SOE ωRNA -HDV-F:	TTATTGCCTAGTATGTCGACGGCCGGCATGGTCCCAGCCTCCTC GCTGGCGCCGGCTG
SOE ωRNA Xho I+HDV-R:	GGCTCGAGGTCCCATTCGCCATGCCGAAGCATGTTGCCCAGCCG GCGCCAGCGAGGAG
TnpB-D187A-F	TTGGAATAGCCCTAGGAGTGGATAAACTAG
TnpB-D187A-R	CACTCCTAGGGCTATTCCAACCACTTTCCC
TnpB-E271A-F	TATACGTGGCAGATCTTGATGTAAAGGATA
TnpB-E271A-R	ATCAAGATCTGCCACGTATATCTCATCATA
TnpB-D355A-F	AGATCGTGCCTATAATGCTTCTCTAAATAT
TnpB-D355A-R	AAGCATTATAGGCACGATCTACAGTCCAAC
**Oligonucleotides for dsDNA and ssDNA**	
DNA-Target-F(5'FAM)	AGCTTTATATTAGAGATAGCATTCATACATACTAGGCAATAAAA ACCGTTAAAGCATCC
DNA-Target-R(5'FAM)	GGATGCTTTAACGGTTTTTATTGCCTAGTATGTATGAATGCTATC TCTAATATAAAGCT
DNA-target-NoTAM-F(5'FAM)	AGCTTTATATTAGAGATAGCATTCATACATACTAGGCAATAAAA ACCGAATTTGCATCC
DNA-target -NoTAM-R(5'FAM)	GGATGCAAATTCGGTTTTTATTGCCTAGTATGTATGAATGCTAT CTCTAATATAAAGCT
DNA-NonTarget-F(5'FAM)	CTAGGGCGCGGCTCTCGCTACGGACGCACGCAGCTTACCGCCCC CAATGGCCCTACGAA
DNA-NonTarget-R(5'FAM)	TTCGTAGGGCCATTGGGGGCGGTAAGCTGCGTGCGTCCGTAGCG AGAGCCGCGCCCTAG
**Primers for construction of target plasmids**	
pTarget-F-NdeI	TATGTTTAACGGTTTTTATTGCCTAGTATG
pTarget-R-SalI	TCGACATACTAGGCAATAAAAACCGTTAAACA
pTarget-F-NoTAM-NdeI	TATGAAATTCGGTTTTTATTGCCTAGTATG
pTarget-R-NonTAM-SalI	TCGACATACTAGGCAATAAAAACCGAATTTCA
**Primers for seed and TAM sequence variation**	
Mut1~5-F	TATGTTTAAGCCAATTTATTGCCTAGTATG
Mut1~5-R	TCGACATACTAGGCAATAAATTGGCTTAAACA
Mut2-F	TATGTTTAACGGTTAAATATGCCTAGTATG
Mut2-R	TCGACATACTAGGCATATTTAACCGTTAAACA

Mut3-F	TATGTTTAACGGTTTTTATACGGAAGTATG
Mut3-R	TCGACATACTTCCGTATAAAAACCGTTAAACA
Mut4-F	TATGTTTAACGGTTTTTATTGCCTTCATAG
Mut4-R	TCGACTATGAAGGCAATAAAAACCGTTAAACA
Mut-5~-1-F	TATGAAATTCGGTTTTTATTGCCTAGTATG
Mut-5~-1-R	TCGACATACTAGGCAATAAAAACCGAATTTCA
TAM-5A-F	TATGATTAACGGTTTTTATTGCCTAGTATG
TAM-5A-R	TCGACATACTAGGCAATAAAAACCGTTAATCA
TAM-5C-F	TATGCTTAACGGTTTTTATTGCCTAGTATG
TAM-5C-R	TCGACATACTAGGCAATAAAAACCGTTAAGCA
TAM-5G-F	TATGGTTAACGGTTTTTATTGCCTAGTATG
TAM-5G-R	TCGACATACTAGGCAATAAAAACCGTTAACCA
TAM-4C-F	TATGTCTAACGGTTTTTATTGCCTAGTATG
TAM-4C-R	TCGACATACTAGGCAATAAAAACCGTTAGACA
TAM-4G-F	TATGTGTAACGGTTTTTATTGCCTAGTATG
TAM-4G-R	TCGACATACTAGGCAATAAAAACCGTTACACA
TAM-4A-F	TATGTATAACGGTTTTTATTGCCTAGTATG
TAM-4A-R	TCGACATACTAGGCAATAAAAACCGTTATACA
TAM-3G-F	TATGTTGAACGGTTTTTATTGCCTAGTATG
TAM-3G-R	TCGACATACTAGGCAATAAAAACCGTTCAACA
TAM-3C-F	TATGTTCAACGGTTTTTATTGCCTAGTATG
TAM-3C-R	TCGACATACTAGGCAATAAAAACCGTTGAACA
TAM-3A-F	TATGTTAAACGGTTTTTATTGCCTAGTATG
TAM-3A-R	TCGACATACTAGGCAATAAAAACCGTTTAACA
TAM-2C-F	TATGTTTCACGGTTTTTATTGCCTAGTATG
TAM-2C-R	TCGACATACTAGGCAATAAAAACCGTGAAACA
TAM-2G-F	TATGTTTGACGGTTTTTATTGCCTAGTATG
TAM-2G-R	TCGACATACTAGGCAATAAAAACCGTCAAACA
TAM-2T-F	TATGTTTTACGGTTTTTATTGCCTAGTATG
TAM-2T-R	TCGACATACTAGGCAATAAAAACCGTAAAACA
TAM-1C-F	TATGTTTACCGGTTTTTATTGCCTAGTATG
TAM-1C-R：	TCGACATACTAGGCAATAAAAACCGGTAAACA
TAM-1G-F：	TATGTTTAGCGGTTTTTATTGCCTAGTATG
TAM-1G-R：	TCGACATACTAGGCAATAAAAACCGCTAAACA
TAM-1T-F	TATGTTTATCGGTTTTTATTGCCTAGTATG
TAM-1T-R	TCGACATACTAGGCAATAAAAACCGATAAACA
Mut1-F	TATGTTTAAGGGTTTTTATTGCCTAGTATG
Mut1-R	TCGACATACTAGGCAATAAAAACCCTTAAACA
Mut2-F	TATGTTTAACCGTTTTTATTGCCTAGTATG
Mut2-R	TCGACATACTAGGCAATAAAAACGGTTAAACA
Mut3-F	TATGTTTAACGCTTTTTATTGCCTAGTATG
Mut3-R	TCGACATACTAGGCAATAAAAAGCGTTAAACA
Mut4-F	TATGTTTAACGGATTTTATTGCCTAGTATG

Mut4-R	TCGACATACTAGGCAATAAAATCCGTTAAACA
Mut5-F	TATGTTTAACGGTATTTATTGCCTAGTATG
Mut5-R	TCGACATACTAGGCAATAAATACCGTTAAACA
Mut6-F	TATGTTTAACGGTTATTATTGCCTAGTATG
Mut6-R:	TCGACATACTAGGCAATAATAACCGTTAAACA
Mut7-F	TATGTTTAACGGTTTATATTGCCTAGTATG
Mut7-R	TCGACATACTAGGCAATATAAACCGTTAAACA
Mut8-F	TATGTTTAACGGTTTTAATTGCCTAGTATG
Mut8-R	TCGACATACTAGGCAATTAAAACCGTTAAACA
Mut9-F	TATGTTTAACGGTTTTTTTTGCCTAGTATG
Mut9-R	TCGACATACTAGGCAAAAAAAACCGTTAAACA
Mut10-F	TATGTTTAACGGTTTTTAATGCCTAGTATG
Mut10-R	TCGACATACTAGGCATTAAAAACCGTTAAACA
**Primers for DNA targeting and genome editing in bacterial cells**	
pMG36e-0632-F	ATCCTCTTCATCCTCTTCGTCTTGGCCCCTTCAGCTTGAGCTCGT
pMG36e-0632-R	CGATCGACCCATATTTAAAAAGCTAGCTATAACACCAGCTTGCTC
PlacZ-SOE36e-F	ATCCTCTTCATCCTCTTCGTCTTGGCCCCTTCAGCTTGAGCTCGT
PlacZ-SOE0632-R	TGGTGGTGATGGTGATGCATAATATCCATTCCCTTCATTT
pMG36e-inverse-TEDA-0632-F	TAGCTTTTTAAATATGGGTCGATCG
pMG36e-inverse-TEDA-0632-R	CCAAGACGAAGAGGATGAAGAGGAT
pMG36e-inverse-TEDA-ωRNA-F	GCGAATGGGACTGCAGTGCATGAGAGCAAAAAAGAGGAGCCG
pMG36e-inverse-TEDA-ωRNA-R	GTCCTTCTTAATCTAGAGCATCTAGAGGATCGATCCCCGGGC
ωRNA-F	TGCTCTAGATTAAGAAGGAC
SOEguide1-ωRNA-R	CCGATCACCGTTGCCGTCGGGTGAACAACCCCGCCCTTAC
SOEguide1+HDV-F	CCGACGGCAACGGTGATCGGGGCCGGCATGGTCCCAGCCTCCTCGCTGGCGCCGGCTGG
HDV-R	TGCACTGCAGTCCCATTCGCCATGCCGAAGCATGTTGCCCAGCCGGCGCCAGCGAGGAG
SOEguide2-ωRNA-R	TTGCGTAGCTAAATGATCAAGTGAACAACCCCGCCCTTAC
SOEguide2+HDV-F	TTGATCATTTAGCTACGCAAGGCCGGCATGGTCCCAGCCTCCTCGCTGGCGCCGGCTGG
pyrEdonor-Left-F	GGCTGGGGGGCATGGACGTC
pyrEdonor-Right-R	TTTCAGACTTTGCAAGCTTACCCGCAAAGTAGACTTGTG
guide@native plasmid-F	CGCCCTAAGAAGCAATGAGAGGCCGGCATGGTCCCAGCCT
guide@native plasmid-R	TCTCATTGCTTCTTAGGGCGGTGAACAACCCCGCCCTTACGGACG
F1	TTATTCGGGTTGTTTGGCCG
R1	TTGGACTTTTCCACAGCCCA

F2	TTAATGACGTAAATTAAGCAAGAAGATCTCG
R2	ATGAGCACCACTATTTTATCATTCCA
F3	TTGGCAGTTAAGAGTATTGGATCAGG
R3	TCACAAACATTGAACATTTTTAGCCG
pyrE-verification-F	TGAGCAGTTACAAAACGCGCT
pyrE-verification-R	GGCCGATAAGTACCGTAGTCAGC
pSeSD-cas6-F	GAATGAGGTGAAGCTCAATGTGGAGAGCCAAGGAGAA
pSeSD-cas6-R	TTACGGTTATAACTCATATCACGATGGAGATTTCCTGC
RNAcas6L-F	ATTTGGCATTCTTTCTAATGCGTGGTGAGTATTTATACCATTT
Cas6r-R	GGTGATGATGATGTGCTATTATTACTTTGATCTTTTATAT
7d-F(NdeI)	TATGAGTTATAACCGTAAAGTATATTTAAGAGAGTAACATAAG GATAACTTGAAGAG
7d-R(SalI)	TCGACTCTTCAAGTTATCCTTATGTTACTCTCTTAAATATACTTT ACGGTTATAACTCA
7D-TEDA-RNA-F	ATAAGGATAACTTGAAGAGTTAAGAAGGACTTGACTTTGGCT
7D-TEDA-RNA-R	GGTATAAATACTCACCACGCATTAGAAAGAATGCCAAATGTGA ACAACCCCGCCCTTAC
cas6-verification-F	GGCTTTGGATAGCTAGAAAG
cas6-verification-R	ACTTGGGCGGAATCTATTAG
